# The impact of crop specialization on nutritional intake: Evidence from farm households in China

**DOI:** 10.1371/journal.pone.0272347

**Published:** 2022-08-05

**Authors:** Fei Sun, Peng Qian, Shouhui Cao, Yuping Chen, Ziyue Feng

**Affiliations:** 1 Center for Social Survey Research, Xuzhou Administration Institute, Xuzhou, China; 2 School of Business Administration, Zhongnan University of Economics and Law, Wuhan, China; 3 School of Economics, Zhongnan University of Economics and Law, Wuhan, China; Sichuan Agricultural University - Chengdu Campus, CHINA

## Abstract

**Background:**

In the wake of the severe impact of COVID-19 on the food security of the vulnerable groups in rural areas, the issue of how to achieve the Sustainable Development Goals 2 aims to “Zero Hunger” (SDG 2) and ensure the food safety of farmers has drawn unprecedented attention. Nutritional intake is generally used as an important indicator to reflect family food security. Under the background that Chinese farmers have gradually changed from the traditional diversified production mode to the specialized production of crops, the main purpose of this article is to explore what are the impact of crop specialization on farmers’ nutritional intake? Could the specialization of crop production be taken as an important measure to ensure the food safety of farmers and achieve the SDG 2?

**Methods:**

Based on the micro-survey data from 866 farmer households in China, this paper using Seemingly Unrelated Regressions model, Group Regression model and Mediating Effect model to analyze the average and heterogeneous effects of crop specialization on the nutritional intake of farmers, as well as the mediating effect of income. In addition, robustness test and endogenous treatment were performed by using alternative explanatory variables and IV-2SLS method was used to estimate the results.

**Results:**

After correcting for endogenous bias, crop specialization had a significant negative impact on energy intake and fat intake of farmers at the statistical level of 5% and 1% respectively, especially for farmers in mountainous areas. Household income played a mediating effect on the effect of crop specialization on farmers’ energy and fat intake, and the proportion of the masking effect was 8.43% and 8.96% respectively. In addition, household financial capital and social capital have a significant positive impact on farmers’ nutritional intake.

**Conclusions:**

Crop specialization cannot guarantee the food safety of farmers in terms of nutritional intake. However, when the development trend of crop specialization is irreversible, more attention should be paid to improving the level of various livelihood capital of farmers, especially those in mountainous areas, and to continuously increasing their income to ease and ultimately eliminate the negative impact of crop specialization on farmers’ nutritional intake, which finally make everyone realize the SDG 2.

## 1. Introduction

In response to the severe challenges faced by the sustainable development of mankind, the 193 member states of the United Nations in 2015 formulated the 2030 Sustainable Development Goals (SDGs), which are comprehensive and universally applicable, and outlined the grand vision for the path to sustainable development in the world [1]. China has always appreciated the SDGs highly, which are reflected in the China’s medium and long-term development strategy based on its specific national conditions. For instance, “Outline of the 14th Five-Year Plan (2021–2025) for National Economic and Social Development and Vision 2035 of the People’s Republic of China” clearly states that it is necessary to actively implement the United Nations 2030 Agenda for Sustainable Development, which provides basic guidelines for the promotion of China’s sustainable development. In February 2021, General Secretary Xi Jinping solemnly declared to the world at the National Poverty Alleviation Summary and Commendation Conference that “China has achieved a comprehensive victory in the fight against poverty”, which means that China has achieved the first goal of the SDGS 10 years ahead of the schedule. The second goal of the SDGs is “end hunger, achieve food security and improve nutrition and promote sustainable agriculture.” (referred to as “Zero Hunger”, SDG 2). At present, following the severe impact of COVID-19 on the food security of the world’s population, especially the rural vulnerable groups, the issue of how to achieve the SDG 2 and ensure the food security of farmers has received unprecedented attention and concern [2–4].

As far as China is concerned, agriculture and rural areas reflect the biggest shortcoming of China’s economic and social development at the present stage, and farmers are faced with severer food insecurity [5]. The World Bank pointed out earlier that farmers’ crop production strategies are closely related to their family food security [6]. Nutritional intake is generally used internationally as an important indicator to reflect family dietary quality and food security [5, 7, 8]. In the broad sense, crop production strategies mainly include crop production specialization and the corresponding crop production diversification, among which crop production specialization is the micro embodiment of agricultural specialization. With the deepening of specialized division of labor in China’s agricultural production field and the improvement of the marketlization level of agricultural products, most of the farmers gradually shifted from the traditional diversified production mode of "small and complete" to the specialized production of crops [9–11]. In terms of relevant policies, the “Opinions on Promoting the Organic Connection of Small Farmers and Modern Agriculture Development”, the “Strategic Plan for Rural Revitalization (2018–2022)”, issued by the state, and the “Law of the People’s Republic of China on the Promotion of Rural Revitalization” pointed out that it is necessary to actively guide small farmers and new agricultural business entities to carry out specialized production and management, allocate production factors in a rational manner, and form a development pattern of one village with one product, one township with one special area, and one county with one industry. It is an undeniable fact that according to the views of classical economics and traditional agricultural economic theory, crop production specialization can improve farmers’ production efficiency and income through translating the principle “practice makes perfect" into the real world [12, 13]. Then, in the context of the transition to specialization in Chinese agriculture, the following question is worth contemplating: What is the impact of crop specialization on the nutritional intake of farmers? Could crop production specialization be taken as an important measure to ensure farmers’ food security and achieve the SDG 2? These are also the key question that this paper attempts to answer.

At present, China has fully realized the poverty alleviation goal with “don’t worry about food” as the core, but having enough food cannot always ensure the nutrition and health of residents, and hunger in the midst of plenty also occurs occasionally [14, 15], which can be addressed by letting a balanced and diverse diet [16]. Ellis pointed out earlier that devising diversified production models is an ideal strategy for small farmers in developing countries to ensure their family survival when facing severe external shocks [17, 18]. As well as the reality that in developing countries such as Thailand, Zambia, and Bangladesh, subsequent production diversification was adopted as a national agricultural development strategy [19–21], which made the academic community more focused on the impact of crop production strategies on nutritional intake of farmers from the perspective of diversification of crop production. Therefore, two types of crop production strategies were considered in this paper to review the literatures on the effects of specialization and diversification of crop production on nutritional intake of farmers.

Jones (2017) investigated the effects of crop production diversification on nutritional intake of farmers based on the data of 3,000 households in Malawi from 2010 to 2013, and the results indicated that the improvement of crop production diversification could significantly increase farmers’ intake levels of energy, protein, iron, vitamin A and zinc [22]. The World Bank (2018) further pointed out that diversified production strategies can protect agricultural production systems from the impacts of climate and market changes, and improve farmers’ intake of nutrients such as protein, vitamins and minerals to enhance their nutritional health [23]. However, Mukherjee (2015) analyzed the relationship between crop production diversification and the per capita intake of energy, protein, fat and other nutrients of farmers based on the survey data of 6 villages in 3 districts of West Bengal in India, and found that the diversification of crop production has a negative correlation with the per capita intake of energy, protein and fat in each region [8]. Argyropoulou (2016) using the survey data of rural children in northern Ghana, showed that there was no correlation between the diversification of crop production and nutritional intake of children [24]. In addition to that, a study based on household survey data collected from Indonesia, Kenya and Uganda found that the different relationships between crop production diversification and household intake of nutrients such as energy, iron, zinc and vitamin A were affected by the study area and the diversity measurement method [25].

In general, there have been many studies of the relationship between crop production specialization (or diversification) and nutritional intake of farmers, which provide important references for this paper, but there remain some shortcomings. First of all, as there are major differences among the research conclusions of the literature based on different countries’ or different farmers’ micro data, an agreement on the optimal strategy has not yet been reached. As pointed out by some scholars, the current research results based on the relationship between crop production specialization or diversity and farmers’ food security are mixed and vary according to the environment [26]. Secondly, effectively guaranteeing the nutritional intake of Chinese residents, especially rural residents, who account for a large proportion of the world’s population, can play a significant role in achieving the Development Goal of “Zero Hunger” at the global level. However, in the context of the rapid transformation of China’s agriculture toward specialization, there is still a lack of literature on the effect of crop production specialization on nutritional intake of farmers based on the survey data collected from Chinese farmers.

Therefore, this paper will supplement the existing literature from the following three aspects: (1) Using the micro-survey data of farmers from the Hubei Rural Survey Team of the National Statistics Bureau of China (NSBC) to study the impact of crop specialization on the nutritional intake of farmers, so as to make up for the lack of sample selection in the existing literature. (2) Introducing farmer income as a mediating variable to construct a theoretical analytical framework including crop production specialization, farmer income, and farmer nutritional intake, and then analyze the internal influence mechanism and path of farmer’s nutritional intake by crop production specialization. (3) Taking the increasingly uneven development between regions and groups in China in the context, this paper further grouped farmers according to the topographic features of the village, and used the Group Regression model to explore the realistic scenarios of the difference in the impact of crop production specialization on the nutritional intake of different groups within farmers. This paper, through its research, can provide a decision-making reference for the scientific promotion of the specialized development process of crop production in China and other countries, ensuring the food security of farmers and taking the lead in realizing the SDG 2.

The paper is structured as follows: After this introduction, the research hypothesis is put forward at first through a theoretical analysis, and then the econometric model is introduced in Section 2. After that, the data sources and core variables are focused on in Section 3. In Section 4, the results of the econometric estimation are presented. The final section provides a summary of the main results and discussion.

## 2. Theoretical analysis

According to the theory of dietary nutrition, there are more than 40 essential nutrients necessary to maintain the normal growth and various physiological activities of the human body. All these essential nutrients must be obtained from food, and no single type of food can satisfy all the energy and nutrients required by the human body, and there is a significant positive correlation between nutritional intake and dietary diversity [27, 28]. Based on this, “food diversity” has become the basic principle of a balanced diet and the core guiding principle of the “Dietary Guidelines for Chinese Residents”.

Simultaneously, according to the analytical framework of sustainable livelihood theory proposed by the Department for International Development (DFID), livelihood strategies affect livelihood outcomes [29], in the context of this paper, means that the strategy taken for the crop production specialization will directly or indirectly affect the nutritional intake, which is one of the livelihood outcomes of farmers. This impact is clearer in the case of small farmers, who use crop production and operations as their main source of livelihood. The Department of Agriculture and Rural Development of the World Bank further pointed out that the production of food crops for direct family consumption is the main way in which agricultural production activities affect the food security of farmers [6], as farmers still consume their directly produced crops to a large extent [30]. However, the constraints of established resource endowments (such as labor, land) set a limit on the scope of production activities that farmers can engage in. The input of crops produces a “crowding out effect”, which, in turn, reduces the types of food that farmers produce for direct consumption by the family and their nutritional intake. In other words, the lack of a diversified agricultural production system will lead to a reduction in the nutritional intake of farmers [22]. From this, it can be concluded that the increase in the level of specialization of crop production will reduce the nutritional intake of farmers.

Moreover, the Department of Agriculture and Rural Development of the World Bank also pointed out another way in which agricultural production affects the food security of farmers, that is, the income earned from selling agricultural products affects the purchase of family food [6], and this should be promoted by crop specialization. An increase in the income of farmers is a prerequisite [31]. Then the question that arises is: ‘Can crop specialization promote the increase of farmers’ income?’ Drawing lessons from the analytical framework of New Classical Economics [12, 32], we assume that farmers mainly engaged in crop production produce only two types of crops *x* and *y*, and the production function of farmers is set as:

$$x^{p}=x^{d}+x^{s}=S_{x}^{a}$$ (1)

$$y^{p}=y^{d}+y^{s}=S_{y}^{a}$$ (2)

In the formula, *x*^*p*^ and *y*^*p*^ are the total planting income of each crop of farmers; *x*^*d*^ and *y*^*d*^, *x*^*s*^ and *y*^*s*^ represent the part of farmers used for self-sufficiency and sales, respectively. *S*_*x*_ and *S*_*y*_ represent the planting area of each crop, which is defined as the production specialization level of farmers in this crop; *a* is the parameter of specialized economic degree $(a>1);\;S_{x}^{a}$ and $S_{y}^{a}$ represent the specialized income of the farmers from specialized production of crops *x* and *y*, respectively. Assuming that the total share of farmers’ crop planting area is 1, the crop planting constraint (i.e. the constraint of farmers’ land factor endowment) is:

$$S_{x}+S_{y}=1$$ (3)

In this case, Eqs (1~3) are referred to as farmers’ agricultural production system. It can be proved that:

$$\frac{dx^{p}}{dS_{x}}=aS_{x}^{a-1}>0,\;\frac{d^{2}x^{p}}{dS_{x}^{2}}=a\left(a-1\right)S_{x}^{a-2}>0$$ (4)

$$\frac{dy^{p}}{dS_{y}}=aS_{y}^{a-1}>0,\;\frac{d^{2}y^{p}}{dS_{y}^{2}}=a\left(a-1\right)S_{y}^{a-2}>0$$ (5)

In the formula, $\frac{dx^{p}}{dS_{x}}$ and $\frac{dy^{p}}{dS_{y}}$ are the marginal income of crop *x* and *y*, respectively. The marginal income is greater than 0 means that both *x*^*p*^(*S*_*x*_) and *y*^*p*^(*S*_*y*_) are increasing functions, that is, planting income of crop increases with the improvement of production specialization level. The second derivative of *x*^*p*^ with respect to *S*_*x*_ and the second derivative of *y*^*p*^ with respect to *S*_*y*_ are still greater than 0, which means that marginal revenue also increases with the improvement of the specialization level of crop production. In terms of average return *AR*_*i*_:

$$AR_{x}=\frac{x^{p}}{S_{x}}=S_{x}^{a-1}\text{,}\;\frac{dAR_{x}}{dS_{x}}=\left(a-1\right)S_{x}^{a-2}>0$$ (6)

$$AR_{y}=\frac{y^{p}}{S_{y}}=S_{y}^{a-1},\;\frac{dAR_{y}}{dS_{y}}=\left(a-1\right)S_{y}^{a-2}>0$$ (7)

It can be viewed in formulas (6) and (7) that the average return will also increase with the increase in the level of specialization of farmer’s crop production. In summary, crop specialization can promote the increase in farmers’ income level. When the level of specialization in crop production by the farmers continues to enhance, they will earn more income to buy various types of food from the agricultural product market. This income conversion process can form a “substitution effect” for the part of the food that farmers give up because of specialized production, so as to continue to maintain and increase their intake of various nutrients.

**Fig 1:**
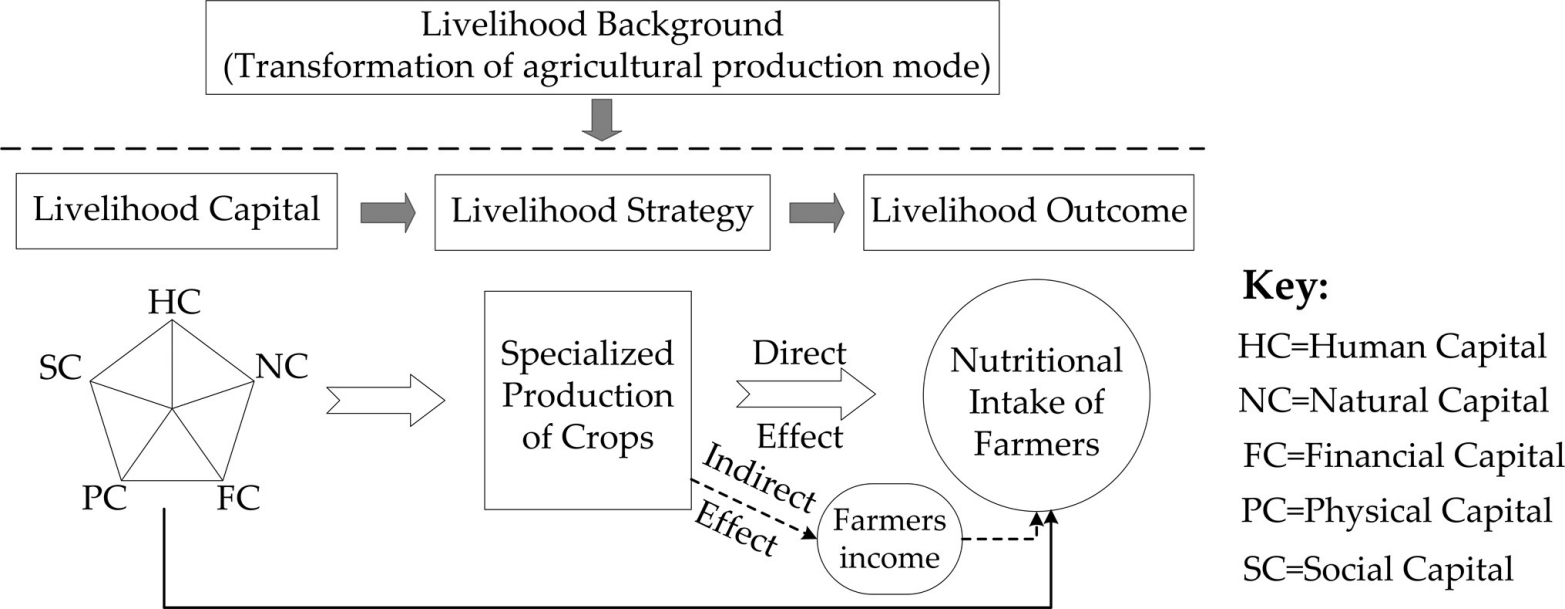
The theoretical framework of this study.

Based on the above analysis of the “crowding out effect” and “substitution effect” of the crop specialization on the number of food types that farmers consume, the following theoretical model (Fig 1) and hypotheses can be drawn:

**Hypothesis 1 (H1).** *The impact of the crop specialization on the nutritional intake of farmers is uncertain*.
**Hypothesis 2 (H2).** *Crop specialization can indirectly affect farmers’ nutritional intake by increasing farmers’ income*.

## 3. Model, data and variables

### 3.1. Model design

This paper focuses on the impact of crop specialization on the intake of three macronutrients of protein, fat, and carbohydrate and the intake of energy converted from these macronutrients by farmers. In this case, multiple equations coexist in the regression analysis. Moreover, it is taken into consideration that the regression equations with different nutritional intakes as the explained variables have common constraints (including income budget constraints, and individual nutritional intakes have the maximum and minimum range constraints), and observed variables such as livelihood capital of farmers or non-observed variables of farmers may be related to the influence of nutrition intake. This allows regression equations to have “cross-equation restrictions” in theory, meaning that the perturbations between different equations are correlated. In this case, using the method of Seemingly Unrelated Regression Estimation (SURE) wherever possible can improve the accuracy of the model’s estimates. The standard model form of SURE is as follows:

$$N\equiv \left(\begin{array}{c} N_{1}\\ N_{2}\\ N_{3}\\ N_{4} \end{array}\right)=\left(\begin{array}{c} HI_{1}\;0\;0\;0\\ \;0\;HI_{2}\;0\;0\\ \;0\;0\;HI_{3}\;\;0\\ \;0\;0\;0\;HI_{4} \end{array}\right)\times \left(\begin{array}{c} \beta_{1}\\ \beta_{2}\\ \beta_{3}\\ \beta_{4} \end{array}\right)+\left(\begin{array}{c} \varepsilon_{1}\\ \varepsilon_{2}\\ \varepsilon_{3}\\ \varepsilon_{4} \end{array}\right)=S\beta +\varepsilon$$ (8)

The economic model of a single nutritional intake is:

$$N_{ij}=\alpha_{0}+\alpha_{1}HI_{i}+\sum_{k=1}^{5}\alpha_{k+1}C_{i}^{k}+\varepsilon_{i}$$ (9)

At the same time, in order to verify whether the income of farmers has a mediating effect on the process of crop production specialization affecting farmers’ nutritional intake, the following mediating effect model was extended on the basis of formula (9):

$$NC_{i}=\beta_{0}+\beta_{1}HI_{i}+\sum_{k=1}^{5}\beta_{k+1}C_{i}^{k}+\mu_{i}$$ (10)

$$N_{ij}=\gamma_{0}+\gamma_{1}HI_{i}+\gamma_{2}INC_{i}+\sum_{k=1}^{5}\gamma_{k+2}C_{i}^{k}+\varphi_{i}$$ (11)

In the above model, *N*_*ij*_(*j* = 1~4) is the explained variable, which respectively represents the intake of energy, protein, fat and carbohydrate of the *i*-th farmer, and *HI*_*i*_ represents the level of crop specialization of the *i*-th farmer. *INC*_*i*_ is the mediating variable of farm household income, which expresses rural households’ disposable income. *α*_0_ ~ *α*_*k*+1_(*k* = 1~5), *β*_0_ ~ *β*_*k*+1_(*k* = 1~5) and *γ*_0_ ~ *γ*_*k*+2_(*k* = 1~5) are the parameters to be estimated, and *ε*_*i*_, *μ*_*i*_ and *φ*_*i*_ are the random error terms of the model. $C_{i}^{k}\left(k=1\sim 5\right)$ is human capital, natural capital, physical capital, financial capital and social capital in the sustainable livelihood theoretical analysis framework of DFID’s.

### 3.2. Data sources and sample selection

In this paper, the rural survey data collected from 56 counties and cities in Hubei Province in 2016 by the Hubei Rural Survey Team of the National Statistics Bureau of China were used. In this survey, stratified random sampling method and daily bookkeeping method were utilized to collect data. When selecting farmers, 1 to 7 villages in each county and 8 to 12 households in each village were selected. In 2016, a total of 2,564 farmers were investigated, and a wide range of data indicators were collected, which could provide rich data support and quality assurance for this study. Compared with other relevant research data, this survey data can not only effectively avoid the problem of “seasonal deviation” caused by the cyclical characteristics of agricultural production [33], but also solve the problem of “recall deviation” in a better way, when using 24-hour review method or food frequency method to collect data.

Since the survey is based on the statistical caliber of China’s national population census, all rural households in the region are contained in the sample frame for sampling. Among them, farmers who are no longer engaged in crop production activities are also under investigation. It is undeniable that skilled farmers in China can get more income by engaging in non-agricultural industries in cities and towns, so a large number of farmers lease all or part of their farmland to others for production. However, the main purpose of this paper is to explore the impact of farmers’ livelihood strategies on livelihood outcomes, i.e. the impact of crop specialization on farmers’ nutritional intake (Fig 1), there is no relationship between crop specialization or diversification and household nutritional intake for these farmers who are no longer primarily engaged in agriculture.

**Fig 2:**
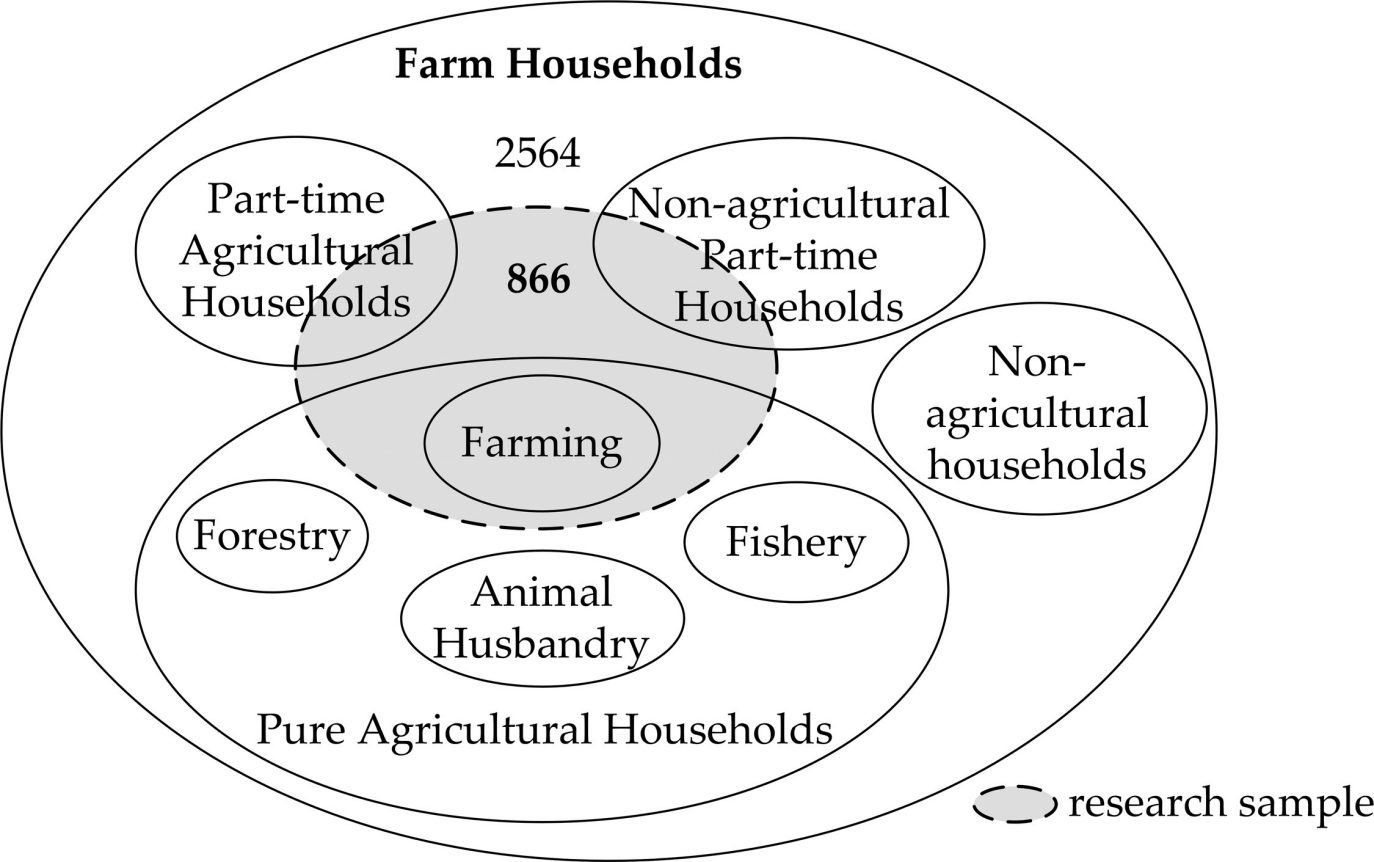
Selection of research sample.

Therefore, in order to accurately identify the net effect and mechanism of crop specialization on the nutritional intake of farmers, according to the classification method adopted by the Ministry of Agriculture and Rural Affairs of China which divides the farm households into four categories (1. Pure Agricultural Households, i.e., pure farmers, which refer to rural households whose income from primary industry accounts for more than 80% of their net household income; 2. Part-time Agricultural Households, also known as I part-time households, refer to rural households whose income from the primary industry accounts for 50% to 80% of the household’s net income; 3. Non-agricultural Part-time Households, namely II part-time households, refer to rural households whose primary industry income only accounts for 20%-50% of the family’s net income; 4. Non-agricultural Households refer to rural households whose primary industry income accounts for less than 20% of the family’s net income), we first remove from consideration the Non-agricultural Households in the survey data, and then remove those households whose crop production income is less than 50% of the net income of the family’s primary industry income. After multiple rounds of screening, 866 valid observations sample was finally obtained to use in this article. In addition, we also illustrate the selection of research sample by graphic illustration (Fig 2).

**Fig 3:**
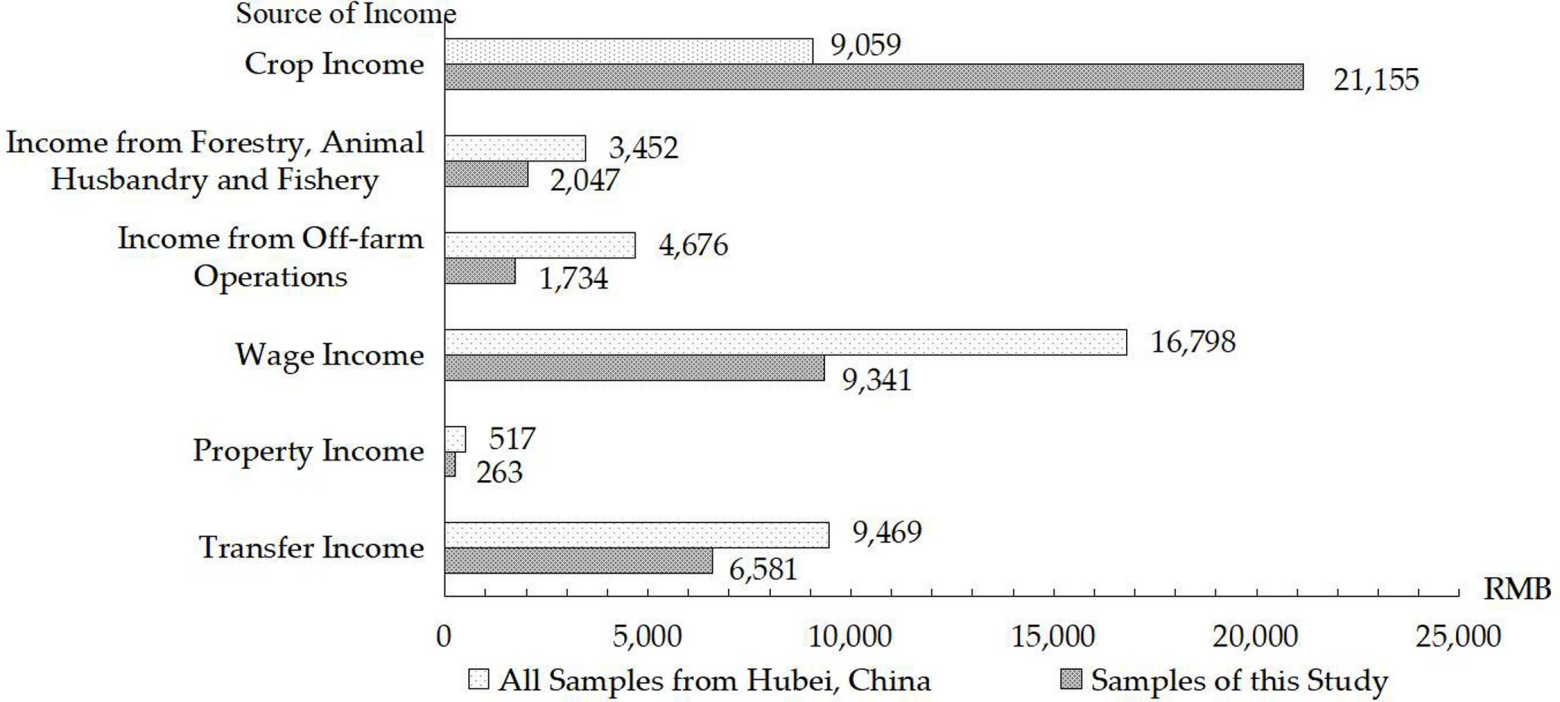
The income structure of the total sample of the province and the sample in this study.

The income structure of the selected 866 effective observations and the total sample of 2,564 rural households in Hubei Province of China is presented in Fig 3. Generally speaking, wage income is the main source of income for the total sample of 2,564 rural households in the province. Farming income from crop production is the main source of income for the 866 sample households taken into consideration in this article, accounting for 51.45% of the total household income, which implies crop production being the main livelihood activity of these farmers in general, and the specific strategies of crop production having a significant effect on their livelihood status.

### 3.3. Variables and descriptive statistics

#### 3.3.1. Dependent variables

The dependent variables in this article are the nutritional intake of the farm household, which, specifically, refers to the daily fat, protein, carbohydrate and energy intake per person of the farmer household. Among them, the human body’s intake of energy is an important indicator of food safety status [34]. The basic data of the food consumed by farmers in this paper came from the micro data collected by NSBC. This survey collected the food data of farmers’ families according to the food category and took the way of accounting. The food included in this study was mainly divided into 10 categories and 40 specific types. These foods are grains (including wheat, rice, corn and other grains, sweet potatoes, potatoes and other potatoes, soybeans and other beans), fats (including vegetable oil and animal oil), vegetables and vegetable products (including fresh vegetables, dried vegetables and vegetable products, fresh mushrooms, dried mushrooms and mushroom products), meat (including pork, beef, lamb and other meats and products), poultry (including chicken, duck, goose and other poultry and products), aquatic products (including fish, shrimps, shells, crabs, algae and other aquatic products), eggs and egg products (including fresh eggs and egg products), milk and dairy products (including fresh milk, yogurt, milk and other dairy products), melons and fruits (including fresh melons, fruit products and nuts), and confectionery and pastries (including sugar, candies, pastries and other confectionery).

However, the “China Food Composition” provided by the Chinese Center for Disease Control and Prevention Nutrition and Health for calculating the nutritional composition of food utilizes the “food category and subcategory”, which differs from the method used by NSBC for food classification. In order to obtain nutritional intake indexes of sample households, a certain scientific standard is required to convert the two sets of data. Referring to existing research practices [35], the conversion formula is set as:

$$N_{ik}=\sum_{i=1}^{N}Q_{i}\times F_{ik},\;F_{ik}=\frac{\sum_{j}^{m}f_{jk}}{m}$$ (12)

In the formula, *Q*_*i*_ is the weight (g) of food *i* eaten by each farmer per person per day. This indicator data is obtained from survey data collected by NSBC. *F*_*ik*_ is the nutrient content *k*(*k* = 1~4, representing protein, fat, carbohydrate and energy, respectively) in Food *i* per g, which is obtained through the summing average of the nutrient content *f*_*jk*_ of subgroup *j* in food *i* in “China Food Composition”.

In addition to that, due to differences between individuals in terms of age, physical condition, labor intensity, and etc., each person’s demand and satisfaction of various nutrients are also different. Therefore, based on the practice of Meng et al. [36], the standard person scale value set by this paper is 0.35 for the population of 0 to 5 years old, 0.5 for the population of 6 to 15 years old, 1 for the population of 16 to 65 years old, and 0.5 for the population of 66 years old and above. The converted household standard of daily intake of fat, protein, carbohydrate and energy is the core dependent variables in this paper.

#### 3.3.2. Independent variables

The core explanatory variable of this paper is crop specialization. The current academic circles generally use the Herfindahl Index (*HI*) to reflect the level of specialization (or diversification) of crop production [19, 37].


$$HI_{i}=\sum_{n_{i}=1}^{N_{i}}{\left(\frac{S_{n_{i}}}{\sum_{n_{i}=1}^{N_{i}}S_{n_{i}}}\right)}^{2}$$ (13)

*HI* represents the professional level of crop production, which is the square sum of the proportion of the sown area $S_{n_{i}}$ of each crop to the total sown area $\sum_{n_{i}=1}^{N_{i}}S_{n_{i}}$ of farmer *i*. The value of *HI*_*i*_ ranges between 0 and 1. The smaller the *HI*_*i*_, the lower the level of crop specialization. If the value is 1, it means that the farmer only grows one kind of crop.

#### 3.3.3. Mediating variables

Farmers’ income is the mediating variable in this study, and this indicator can be obtained directly from farmer survey data. It should be noted that according to the statistical caliber of NSBC, the income of farmers includes cash income (without deduction of production costs), total income (without deduction of production costs), and disposable income. Among them, disposable income is also known as net income, which can reflect the living standards and consumption capacity of farmers in a better way. Therefore, this study adopts the statistical caliber of disposable income.

#### 3.3.4. Control variables

According to the sustainable livelihood theory analysis framework of DFID, this study considered the livelihood capital of farmers as other control variables affecting the nutritional intake of farmers’ livelihood outcomes. Referring to the practice of existing research [29, 38], indicators such as the size of permanent resident population and the education level of the head of the household were selected to represent the human capital owned by the household; the quantity of household durable consumer goods and the type of toilet were selected to represent the physical capital; and the total area of agricultural land operated by the household was selected to represent the natural capital. The financial capital and social capital owned by farm households are represented by the household’s deposit and loan status, and the family’s wedding and funeral gift expenditure respectively.

Explanatory and descriptive statistics for all variables are shown in Table 1. It can be seen that, except for protein intake, the intake of nutrients by the sample farmers fulfilled the Chinese guideline level [35].

**Table 1:** Variables’ definitions and specifications.

Type	Variables	Specification	Symbol	Mean	S.D.	Min	Max
Dependent Variables	Energy intake	Average daily energy intake (kJ/ standard person/day, log)	*EI*	9.261	0.448	7.469	10.745
	Protein intake	Average daily protein intake (g/ standard person/day, log)	*PI*	4.305	0.447	2.246	5.892
	Fat intake	Average daily fat intake (g/ standard person/day, log)	*FI*	4.401	0.554	2.545	6.369
	Carbohydrate intake	Average daily carbohydrate intake (g/ standard person/day, log)	*CI*	5.911	0.526	2.778	7.345
Independent Variable	Crop specialization	Sum of squares of the proportion of the total sown area of each crop	*HI*	0.493	0.22	0.154	1
Mediating Variable	Farmers income	Sum of household income from business, wage, property and transfer (yuan, log)	*INC*	10.396	0.687	7.965	13.186
Control Variables	population size	Total standard population of a farm household	*POP*	2.388	0.892	0.5	5.5
	Education level	Educational level of the head of household: never attended school = 1; primary school = 2; junior high school = 3; high school = 4; junior college = 5; bachelor degree or above = 6	*EDU*	2.749	0.696	1	5
	Number of durable consumer goods	The number of household durable consumer goods such as cars, motorcycles/moped, washing machines, refrigerators, color TV sets (with cable TV), air conditioners, water heaters, mobile phones (with Internet access), computers, and cameras	*DCG*	5.109	1.984	0	10
	The toilet type	Household toilet type: no toilet = 1; ordinary dry toilet = 2; sanitary dry toilet = 3; flushing non-sanitary toilet = 4; flushing sanitary toilet = 5	*TOI*	3.218	1.285	1	5
	Farmland scale	Total area of arable land, forest land, garden land and other agricultural land managed by farmers (ha)	*SCA*	1.322	1.93	0.02	30.015
	Household deposits and loans	Whether households have savings or loans: none = 0, yes = 1	*DL*	0.145	0.353	0	1
	Social spending	Expenditures of favors and gifts for family weddings and funerals (yuan, log)	*SS*	7.526	2.324	0	11.704

## 4. Results and discussion

### 4.1. Baseline results

In this paper, the econometric analysis software of Stata15.0 and SURE estimation method were used to estimate the results of model (8) as shown in Table 2. First of all, it should be noted that the basic assumption of the SURE estimation method is that there is a simultaneous correlation between the disturbance terms of the four nutrients intake equation and the null hypothesis H0 which states: “The perturbation terms of each nutritional intake equation have no temporal correlation” (that is, they are independent of each other). However, the test results show that the Breusch-Pagan empirical *P*-value of *H0* is 0.0000 (*chi*^2^(6) = 2931.829), so the null hypothesis of the perturbation terms of the four nutrients intake equation having no temporal correlation, which is rejected at the significance level of 1%. It means that the disturbance term of the four nutrients intake equation is related, indicating that the SURE estimation method is helpful to improve the estimation efficiency for the systematic estimation of the four nutrients intake equation.

**Table 2:** Effect of crop production specialization on nutritional intake of farmers.

Variables	(1)	(2)	(3)	(4)
	Y = *EI*	Y = *PI*	Y = *FI*	Y = *CI*
*HI*	-0.1892***	-0.1323**	-0.4607***	-0.0964
	(0.0612)	(0.0597)	(0.0776)	(0.0754)
*POP*	-0.2136***	-0.2463***	-0.2223***	-0.2040***
	(0.0157)	(0.0153)	(0.0199)	(0.0193)
*EDU*	-0.0584***	-0.0446**	-0.0530**	-0.0613**
	(0.0200)	(0.0195)	(0.0253)	(0.0246)
*DCG*	-0.0089	-0.0037	-0.0169*	-0.0032
	(0.0075)	(0.0073)	(0.0095)	(0.0093)
*TOI*	-0.0188*	-0.0066	-0.0004	-0.0338**
	(0.0109)	(0.0107)	(0.0139)	(0.0135)
*SCA*	-0.0048	0.0049	-0.0044	-0.0046
	(0.0070)	(0.0068)	(0.0089)	(0.0086)
*DL*	0.1036***	0.1039***	0.0956**	0.1223***
	(0.0382)	(0.0372)	(0.0484)	(0.0470)
*SS*	0.0159***	0.0208***	0.0115	0.0188**
	(0.0061)	(0.0059)	(0.0077)	(0.0075)
*Constant*	10.0022***	4.9427***	5.2970***	6.5857***
	(0.0767)	(0.0748)	(0.0972)	(0.0944)
*Prob>chi* ^*2*^	0.0000	0.0000	0.0000	0.0000
*R* ^2^	0.2303	0.2648	0.1917	0.1544
Breusch-Pagan test of independence: *chi*^2^(6) = 2923.925***				

Note: Standard errors in parentheses; *, **, *** denote significance at the 10%, 5%, and 1% level, respectively.

The estimated results of energy, protein, fat and carbohydrate intake as independent variables are shown in Table 2. It can be seen that crop production specialization has significant negative effects on energy, protein and fat intake at the statistical level of 5% or 1%, but has no statistically significant effect on carbohydrate intake. This indicates that the higher the level of crop specialization, the lower the energy, protein and fat intake of farmers are, which is consistent with the results of Jones [22], suggesting that diversified production strategies may be more conducive to farmers in improving their nutritional intake and enhancing their nutritional health [23].

As far as other control variables are concerned, population size and education level have a significant negative impact on the intake of nutrients such as energy, protein, fat and carbohydrate, that is, the larger the household size and the higher the education level of the head of the household, the lower the intake of energy and various nutrients of the family members is. However, household savings and personal expenses had significant positive effects on the intake of nutrients except fat. As far as household savings and loans, and human expenditures are concerned, which represent the financial capital and social capital of farmers, they have significant positive effects on the intake of energy, protein and carbohydrate at the statistical level of 5% or 1%, indicating that the better the economic conditions of farmers and the more human contacts they have, the higher their nutritional intake is.

### 4.2. Robustness tests

For ensuring the robustness of the above estimated results, this part of the study adopts the method of replacing core explanatory variables with control variables for the purpose of conducting test. Specifically, the *HI* was replaced by the maximization index (*MI*) to measure the specialization level of crop production of the core explanatory variable. The *MI* refers to the proportion of crops to the largest sown area in the total sown area to represent farmers’ crop production specialization level. Simultaneously, considering the possibility of location factors having an impact on nutritional intake of farmers, location factor indicators are further added and controlled. The added location factor index includes travel convenience (i.e. whether the village can take buses conveniently: no = 0; yes = 1), and distance between county and village (distance from this village to the nearest county seat: within 2 *km* = 1; 2–5 *km* = 2; 5–10 *km* = 3; 10–20 *km* = 4; Above 20 *km* = 5). The results of robustness test are shown in Table 3.

**Table 3:** The estimated results of robustness check.

Variables	(1)	(2)	(3)	(4)
	Y = *EI*	Y = *PI*	Y = *FI*	Y = *CI*
*HI*	-0.1226*	-0.0832	-0.4048***	-0.0122
	(0.0646)	(0.0633)	(0.0827)	(0.0790)
*POP*	-0.2108***	-0.2443***	-0.2198***	-0.2003***
	(0.0156)	(0.0153)	(0.0199)	(0.0190)
*EDU*	-0.0650***	-0.0491**	-0.0614**	-0.0689***
	(0.0198)	(0.0194)	(0.0254)	(0.0242)
*DCG*	-0.0076	-0.0032	-0.0166*	-0.0009
	(0.0075)	(0.0073)	(0.0096)	(0.0091)
*TOI*	-0.0155	-0.0050	0.0003	-0.0283**
	(0.0109)	(0.0107)	(0.0140)	(0.0133)
*SCA*	-0.0074	0.0037	-0.0052	-0.0088
	(0.0070)	(0.0069)	(0.0090)	(0.0086)
*DL*	0.0996***	0.1008***	0.0904*	0.1179**
	(0.0378)	(0.0371)	(0.0484)	(0.0462)
*SS*	0.0158***	0.0207***	0.0119	0.0184**
	(0.0060)	(0.0059)	(0.0077)	(0.0074)
*Travel Convenience*	0.0766***	0.0594**	0.0883**	0.0944***
	(0.0286)	(0.0280)	(0.0366)	(0.0349)
*Distance between County and Village*	0.0477***	0.0256*	0.0225	0.0741***
	(0.0136)	(0.0133)	(0.0174)	(0.0166)
*Constant*	9.7205***	4.7775***	5.1671***	6.1479***
	(0.1027)	(0.1007)	(0.1315)	(0.1255)
*Prob>chi* ^*2*^	0.0000	0.0000	0.0000	0.0000
*R* ^2^	0.2444	0.2700	0.1901	0.1812
Breusch-Pagan test of independence: *chi*^2^(6) = 2920.403***				

Note: Standard errors in parentheses; *, **, *** denote significance at the 10%, 5%, and 1% level, respectively.

It can be seen that after replacing the core explanatory variables with control variables, crop production specialization still has a significant negative impact on farmers’ energy and fat intake, and has no statistical significance on farmers’ carbohydrate intake. However, the impact of crop production specialization on farmers’ protein intake also lacks statistical significance, which causes the inconsistent results with benchmark estimates. The benchmark model of setting model to estimate errors is possibly caused by the endogenous problems. In order to obtain the causal effect of crop specialization on the household nutritional intake, this study should solve the endogenous problem.

In order to overcome the estimation bias caused by endogenous, the IV-2SLS estimation method introducing instrumental variables was used to estimate the benchmark model. This paper selected the aggregation data—village-level index of crop production specialization—as an instrumental variable. It is one of the most common ideas to use instrumental variables from regional agglomeration data to solve endogenous problems [39, 40]. The rationality of adopting village-level specialization level of crop production as instrumental variable in this paper lies in the following: According to the Social Homophily Theory or Peer Effect in economics and sociology, farmers’ crop production strategies are selective, and farmers in the same village have tendency to make similar choices. The specialization level of crop production in a village can reflect the production strategy of a family to a certain extent. But the level of crop specialization of other farmers in the same village did not have a direct impact on the family’s nutritional intake.

Before using instrumental variable analysis, the effectiveness of village-level crop production specialization of instrumental variable should be tested, including *Underidentification Test* and *Weak Instrumental Variable Test*. The test results are shown in Table 4 (lower part). It can be seen that the *Kleibergen-Paap rk LM* of *Underidentification Test* is 255.662, which, at the 1% level significance, indicates that there is no unrecognizable problem in the index of crop production specialization level at the village level of the selected instrumental variable.

**Table 4:** The estimated results of IV-2SLS.

Variables	(1)	(2)	(3)	(4)
	Y = *EI*	Y = *PI*	Y = *FI*	Y = *CI*
*HI*	-0.1586**	-0.0794	-0.5213***	-0.0234
	(0.0735)	(0.0718)	(0.0931)	(0.0908)
*POP*	-0.2107***	-0.2431***	-0.2205***	-0.2014***
	(0.0160)	(0.0156)	(0.0203)	(0.0198)
EDU	-0.0598***	-0.0427**	-0.0495*	-0.0650**
	(0.0205)	(0.0200)	(0.0260)	(0.0253)
DCG	-0.0097	-0.0051	-0.0186*	-0.0037
	(0.0077)	(0.0075)	(0.0098)	(0.0095)
TOI	-0.0257**	-0.0126	-0.0034	-0.0433***
	(0.0111)	(0.0109)	(0.0141)	(0.0137)
SCA	-0.0089	0.0018	-0.0121	-0.0074
	(0.0086)	(0.0084)	(0.0109)	(0.0106)
DL	0.1118***	0.1070***	0.1019**	0.1329***
	(0.0389)	(0.0380)	(0.0493)	(0.0481)
SS	0.0161**	0.0211***	0.0114	0.0197**
	(0.0063)	(0.0061)	(0.0079)	(0.0078)
*Constant*	10.0084***	4.9275***	5.3370***	6.5781***
	(0.0820)	(0.0801)	(0.1038)	(0.1013)
*Prob>chi* ^*2*^	0.0000	0.0000	0.0000	0.0000
*R* ^2^	0.2303	0.2616	0.1919	0.1556
Diagnostic checking				
*Kleibergen-Paap rk LM*		255.662***		
*Shea’s Partial R* ^2^		0.7137		
*Robust F*		2518.43		
*Mineval*		2044.19		

Note: Standard errors in parentheses; *, **, *** denote significance at the 10%, 5%, and 1% level, respectively.

*Weak Instrumental Variable Test* found that *Shea’s Partial R*^2^ was relatively high (0.7137) in IV-2SLS first-stage regression, and the value of robust *F* is 2518.43, which was significant at the statistical level of 1%. According to empirical rule of thumb, the null hypothesis of “weak instrumental variables” in crop specialization at village level can be rejected. In addition, since IV-2SLS may cause size distortion in the presence of weak instrumental variables, the *Wald Test* with a nominal normal size of 5% for the significance of crop specialization should be further studied. If the “true size” is below 15%, the null hypothesis that "weak instrumental variables exist in crop production specialization at village level” can be rejected. In this paper, the minimum eigen value Statistic (*Mineval*) is 2044.19. It is much higher than the critical value of 8.96 under the 15% bias (it is also greater than the critical value of 16.38 under the 10% bias). In summary, the selected instrumental variables do not have the problem of unrecognizable and weak instrumental variables, which further proves the rationality of the selection of instrumental variables.

Table 4 (upper part) reports the estimated results of IV-2SLS. It can be observed that after overcoming the endogenous problem, the impact of crop production specialization on farmers’ protein and carbohydrate intake is still not statistically significant, which is consistent with the robustness test results, and is different from the benchmark regression estimation results. Moreover, crop specialization still has a significant negative impact on farmers’ energy and fat intake. Based on the above benchmark regression, robustness test results, and endogenous processing results, it can be observed that crop specialization has a significant negative impact on the energy and fat intake of farmers, while it has a significant negative impact on their protein and carbohydrate intake. The impact of intake is not statistically significant, that is, crop specialization will inhibit the energy and fat intake of farmers.

### 4.3. Analysis of heterogeneity: Estimated by grouping farmers

The previous part of the study corroborated the fact that crop specialization will have a significant negative impact on energy and fat intake of farmers. In the context of the continuous expansion of regional and inter-group development imbalance in China, further clarification on the impact of crop production specialization on the energy and fat intake of different groups within farmers has important referential value for the government to formulate targeted policies and measures. So, in this part, farmers are divided into groups according to village terrain characteristics (including plains areas, hilly areas and mountainous areas) for the purpose of analysis. It should be noted that it lacks the support of statistical test to judge the differential impact of crop production specialization simply by comparing between the estimated value and significance level of coefficient for different farmer groups. Therefore, on the basis of grouping estimation, SUEST method is adopted to test the difference of regression coefficients.

The results of the grouping estimation are shown in Table 5. Among them, columns (1)~(3) respectively represent the impact of farmers’ crop production specialization level on the energy intake of farmers in the plains, hills and mountains areas. It can be seen that crop specialization has no significant impact on farmers in hilly areas, but has a significant negative impact on farmers in plains and mountainous areas at the statistical level of 1%. In terms of the absolute value of the coefficients of each group and the difference between the coefficients of the groups, the estimated coefficient of the mountain farmer group has the largest absolute value, and is significantly different from the estimated coefficient of the farmer group in other regions at the 1% statistical level, which shows that when compared with the plains and hilly areas farmers, crop specialization has a stronger negative effect on the energy intake of farmers in mountainous areas.

**Table 5:** Estimation results of farmers grouping and coefficient difference test.

Variables	Y = *EI*			Y = *FI*		
	(1) Plains Areas	(2) Hilly Areas	(3) Mountainous Areas	(1) Plains Areas	(2) Hilly Areas	(3) Mountainous Areas
*HI*	-0.3496***	-0.0178	-0.4414***	-0.3101***	-0.3722***	-1.1086***
	(0.0755)	(0.0836)	(0.1085)	(0.1092)	(0.1075)	(0.1684)
*Control Variables*	YES	YES	YES	YES	YES	YES
*Constant*	10.1154***	10.1689***	9.8437***	5.4599***	5.4549***	5.1740***
	(0.1060)	(0.1210)	(0.1373)	(0.1372)	(0.1432)	(0.1875)
*Prob>F*	0.0000	0.0000	0.0000	0.0000	0.0000	0.0000
*R* ^2^	0.3774	0.2167	0.4659	0.2813	0.1938	0.3712
*Coefficient Difference Test*	**Groups**		**Test Result**	**Groups**		**Test Result**
	Plains Areas and Hilly Areas		0.07	Plains Areas and Hilly Areas		0.05
			[0.7912]			[0.8297]
	Plains Areas and Mountainous Areas		11.91***	Plains Areas and Mountainous Areas		11.44***
			[0.0006]			[0.0007]
	Hilly Areas and Mountainous Areas		9.10***	Hilly Areas and Mountainous Areas		13.49***
			[0.0026]			[0.0002]

Note: Standard errors in parentheses; Empirical *P*-value of coefficient difference test in square brackets; *, **, *** denote significance at the 10%, 5%, and 1% level, respectively.

The regression results (4)~(6) respectively represent the impact of the level of crop production specialization in the plains, hills and mountain areas on the fat intake of farmers. The results indicates that crop specialization has a significant negative impact on the fat intake of farmers in different areas, and the absolute values of the estimated coefficients of farmers in the plains, hills and mountainous areas show an increasing trend. From the test results of the coefficient differences between groups, it is observed that the estimated coefficient differences between the plains and mountainous areas, and that between hills and mountainous areas reached a significant level of 1%, indicating that crop specialization has a stronger negative effect on the fat intake of farmers in mountainous areas.

Based on the above grouping estimation and the test results of the coefficient difference between groups, it can be observed that compared with plains and hilly areas, crop specialization has a stronger negative effect on the energy and fat intake of farmers in mountainous areas. It shows that in the process of advancing the professional development of crop production, the nutrients and food security of farmers in mountainous areas will face more prominent negative impacts.

### 4.4. Mechanism analysis: The mediating effect of farmers income

The empirical results above show that crop production specialization has a significant negative impact on farmers’ energy and fat intake, and has a stronger negative effect on farmers in mountainous areas. In order to verify the research hypothesis H2, the next step is to explore further into whether farmers’ income plays a mediating role in the effect of crop production specialization on the farmers’ energy and fat intake. The results are shown in Table 6 (the upper part). In addition to that, the estimation results of column (1) and column (2) in Table 2 should be combined for discussion during the analysis.

**Table 6:** The mediating effect test results of farmers income.

Test Method	Variables	(1)		(2)		(3)		
		Y = *INC*		Y = *EI*		Y = *FI*		
*Causal Steps Approach*	*HI*	0.2613***		-0.2491***		-0.4828***		
		(0.0798)		(0.0509)		(0.0706)		
	*INC*	—		0.0492**		0.1382***		
				(0.0215)		(0.0291)		
	*Control Variables*	YES		YES		YES		
	*Constant*	8.8048***		9.6733***		4.2416***		
		(0.1111)		(0.2029)		(0.2723)		
	*Prob>F*	0.0000		0.0000		0.0000		
	*R* ^2^	0.3295		0.3783		0.3410		
*Bootstrapping*	**Effect**	**Y = *EI***				**Y = *FI***		
		**EV**	**LLCI**		**ULCI**	**EV**	**LLCI**	**ULCI**
	*Direct Effect*	-0.1992	-0.3065		-0.0919	-0.4992	-0.6529	-0.3455
	*Indirect Effect*	0.0168	0.0040		0.0393	0.0447	0.0183	0.0847
	*Direct Effect / Indirect Effect*	-0.0843	-0.2511		-0.0174	-0.0896	-0.1757	-0.0335

Note: Standard errors in parentheses; EV = Estimated Value, LLCI = Lower Limits of Confidence Interval, ULCI = Upper Limits of Confidence Interval; *, **, *** denote significance at the 10%, 5%, and 1% level, respectively.

First, without considering the mediating variables, it can be observed from the estimation results of column (1) and column (2) in Table 2 that the regression coefficients *α*_1_ of crop production specialization are statistically significant, so it can be carried into the follow-up test. Table 6 column (1) represents the estimation result of Eq (10). The results show that the regression coefficient *β*_1_ of crop production specialization is significantly positive at the level of 1%, implying that crop production specialization has a significant positive impact on farmers’ income.

Columns (2)~(3) represent the estimation results of Eq (11). The results show that in the model with energy and fat intake as explained variables, *γ*_2_ is significantly positive at 1% level, so the complete mediation effect test can be executed at this time. According to the mediating effect criteria proposed by Baron and Kenn [41], the regression coefficients *γ*_1_ of crop production specialization represented in column (2)~(3) were significantly positive at the levels of 5% and 1%, implying that household income played a partial mediating effect in the effect of crop production specialization on household nutritional intake. The signs of *β*_1_ × *γ*_2_ and *γ*_1_ are opposite, so the mediating effect of farmers’ income here is a masking effect (or called mitigating or inhibiting effect), which means that, farmers’ income can indirectly mitigate the negative impact of crop production specialization on household energy intake and fat intake.

In addition to that, the reasonableness and effectiveness of the Causal Steps Approach are being criticized and questioned more and more in recent years, although it is the most popular analytical method for testing the Causal effects. Some scholars even call for the non-parametric Bootstrapping method with higher test effectiveness to replace the step-up regression method. Therefore, in order to ensure the reliability of the mediation effect test results in this paper, the masking effect of farmers’ income is further tested by the bias correction non-parameter percentile Bootstrapping method.

Specifically, the repeated sampling times are set at 5000 times and the confidence interval is 95%. The estimation results of the non-parametric percentile Bootstrapping method with bias correction are shown in Table 6 (the lower part). It can be observed that in the model with farmers’ energy intake or fat intake as the explained variables, direct effect and indirect effect (i.e., the cover effect) and the ratio of the direct effect and indirect effect of the confidence interval do not contain 0, so we can determine that the farmers’ income through crop production specialization and its negative influence on the household energy and fat intake played a masking effect, namely it makes crop production specialization have a negative impact on farm household energy and fat intake. As can be seen from the absolute value of Estimated Value, masking effect of farmers’ income takes up 8.43% and 8.96% of the direct effects, respectively.

## 5. Conclusions

Based on the theoretical analytical framework of the relationship between crop specialization and farmers’ nutritional intake, this paper empirically studied the average and heterogeneous impact of crop specialization on farmers’ nutritional intake and the mediating effect of income, using the farmers micro survey data collected by NSBC in 2016. The main conclusions are as follows: (1) Crop production specialization has a significant negative impact on farmers’ energy and fat intake after overcoming the endogenous problem of the model, which implies that from the perspective of nutritional intake, crop production specialization is not conducive to improving farmers’ livelihood and welfare. (2) In terms of heterogeneity impact, crop specialization has a stronger inhibition effect on energy and fat intake of farmers in mountainous areas compared with farmers in plain and hilly areas, which indicates that farmers in mountainous areas will face a more serious negative impact on food security in the process of promoting the development of crop specialization. (3) According to the test results of the mediating effect of farmers’ income, farmers’ income played a masking effect in the negative impact of crop production specialization on farmers’ energy and fat intake, and the proportion of the masking effect was 8.43% and 8.96%, respectively. (4) Financial capital and social capital, such as household savings and loans, and human expenditure, have a significant positive impact on the nutritional intake of farmers.

The study, using Chinese sample in this paper, also shows that crop specialization is not conducive to ensuring the food security of farmers at the present stage, as also observed in most developing countries. However, as an important part of the modern economic system, agricultural economy is inevitably involved in a highly open specialized division of labor system with the development of China’s socialization of agricultural production, regional specialization of agriculture and commercialization of agricultural products [10]. The trend of farmers getting rid of the traditional “small and complete” production mode and stepping into the development track of modern agriculture cannot be reversed, and it is no longer feasible to maintain or improve the diversification of crop production [9]. At the policy level, a series of important documents issued by the Chinese government also indicated that we should actively guide small farmers and new agricultural entities to execute specialized production and management, rationally allocate production factors, and form a development pattern of one village with one product, one township with one special production, and one county with one industry. Although crop specialization will decrease the nutritional intake of farmers at present, this paper also confirms that the crop specialization can increase their income and alleviate the negative impact on the nutrition intake, and, the financial capital and social capital, considered as important control variables in this paper, can also improve their nutritional intake and farming income. Therefore, when the trend of crop specialization is irreversible, more attention should be paid to improving the level of various livelihood capital of farmers, and to continuous increasing of their income in order to alleviate and ultimately eliminate the negative impact of crop specialization on farmers’ nutritional intake.

On the other hand, the empirical results indicate that crop specialization has a stronger negative effect on nutritional intake of farmers in mountainous areas. These groups are relatively more vulnerable to the promotion of the rural revitalization strategy and realizing the overall development of farmers in China at the present stage. Therefore, in the context of the transition of crop production to specialization, it is necessary for the society and the government to pay special attention to the food quality and safety of these vulnerable groups. In mountainous areas, as mentioned above, farmers can satisfy their food needs by either producing by themselves or buying food in the market [6]. The convenience and accessibility of the market are the prerequisites for farmers to purchase food in the market to realize food security. Therefore, the spatial layout of farmers’ markets and supermarkets in mountainous areas should be optimized, and the transportation conditions between farmers and supermarkets should be improved, so that farmers in mountainous areas can buy all kinds of food needed by their families in a convenient manner, which finally will make everyone realize the SDG 2.

## 6. Limitation and future research

It should be noted that there are some limitations in this paper. Although this paper puts five types of livelihood capital of farmers as control variables into the model according to the core viewpoint of sustainable livelihood analysis framework, and according to a formal bounding argument method developed by Oster [42], it was verified that the model would not lead to model estimation bias due to the omission of some control variables (S1 Table). However, it turns out that other variables, such as distance to farmers’ markets and traffic conditions, also affect farmers’ nutritional intake and should be taken into account in the future research.

In addition, due to the limitation of data, this paper only uses the survey data from Hubei Province, China, and in the future research should expand the survey area. However, it is undeniable that Hubei province is a typical agricultural province in China [43], with a land area of 18.59 million hectares and a population of 58.3 million, of which plains, hills and mountainous areas account for 20%, 24% and 56% of the total area respectively. In 2021, the urbanization rate of Hubei Province was 64.1%, which is generally consistent with the national average of 64.7%. These typical characteristics of Hubei province make the main research conclusions and policy implications of this paper have important reference significance for other regions in China. At the same time, the overall trend of agricultural economic development is consistent from a worldwide perspective [9, 10], so this paper has reference value for other agriculture-oriented developing countries.

## Supporting information

S1 Table The estimated results of omission variable. (DOCX)
