# Supplementary material for: The impact of crop specialization on nutritional intake: Evidence from farm households in China
Source: PLoS One. 2022 Aug 5;17(8):e0272347. doi: 10.1371/journal.pone.0272347 (PMC9355192; doi:10.1371/journal.pone.0272347)
Supplement: S1 Table — (DOCX) [file pone.0272347.s001.docx]

**S1 Table. The estimated results of omission variables.**

| **Estimator** | **Y= *EI*** | **Y= *FI*** |
| --- | --- | --- |
| *HI* (Other variables were not controlled) | -0.2112***  (0.0689) | -0.4876***  (0.0840) |
| *R*^2^ | 0.0108 | 0.0375 |
| *HI* (Other variables were controlled) | -0.1892***  (0.0616) | -0.4607***  (0.0780) |
| *R*^2^ | 0.2303 | 0.1917 |
| *R*^2^×1.3 | 0.2994 | 0.2492 |
| *HI* (When $\delta$= 1) | -0.1821***  (0.0560) | -0.4505***  (0.0805) |
| $\delta$ (When *HI*= 0) | 20.9306 | 22.5807 |

Notes: Standard errors in parentheses; *, **, *** denote significance at the 10%, 5%, and 1% level, respectively.
